# Metastases to Both Parotid Glands Six and Twelve Years after Resection of Renal Cell Carcinoma

**DOI:** 10.1155/2018/7301727

**Published:** 2018-02-18

**Authors:** Achim M. Franzen, Shirrell Glitzky, Ashok Moganti, Frank Lippek, Annekatrin Coordes

**Affiliations:** ^1^Department of Otorhinolaryngology, Head and Neck Surgery, Ruppiner-Kliniken GmbH, Brandenburg Medical University-Theodor Fontane, Campus Ruppiner Kliniken, Fehrbelliner Straße 38, 16816 Neuruppin, Germany; ^2^Institute of Pathology, Ruppiner-Kliniken GmbH, Brandenburg Medical University, Campus Ruppiner Kliniken, Fehrbelliner Straße 38, 16816 Neuruppin, Germany; ^3^Department of Otorhinolaryngology, Head and Neck Surgery, Charité-Universitätsmedizin Berlin, Campus Benjamin Franklin, Hindenburgdamm 30, 12200 Berlin, Germany

## Abstract

Metastases of renal cell carcinoma (RCC) involving the parotid gland are very rare. We present to our knowledge the first case of a 74-year-old woman with metastases of an RCC which affected both parotid glands six and twelve years following curative therapy.

## 1. Introduction

The incidence of malignant neoplasms of the parotid gland ranges between 20% and 30%. Metastases account for approximately 5–25% of malignant tumours [1]. In most cases, these will be metastases of squamous cell carcinoma of the facial skin and scalp (60%), but more rarely, the primary tumour may originate below the clavicle. Distant metastases of infraclavicular primary tumours are in descending order of frequency, located in the lung, kidney, and breast, respectively, and rarely in the gastrointestinal tract, prostate, and skin below the clavicle [1, 2]. The objective of this case report is to reiterate that a lesion in the parotid gland may represent a metastasis of a primary tumour which may be the first manifestation of an unknown primary cancer or which may have been treated years before. While most of these primary tumours are located above the clavicle and arise from the skin of the face and scalp in most cases, a tumour from an infraclavicular location must be considered, and appropriate staging investigations are recommended.

## 2. Case Report

A 74-year-old female presented with a two-month history of rapidly growing painless mass in the left preauricular region. Her past medical history included curative intent surgery for colorectal carcinoma in 2003 and renal cell carcinoma (RCC) seven years before. The adenocarcinoma of the colon ascendens (TNM stage pT3 N1 (3/22) G2 L1 V0 R0 M0; UICC stage IIIB) was treated with a hemicolectomy. The histopathology of the RCC was of a clear cell type (TNM stage pT1 NX G2 L0 V0 R0 M0) and Fuhrmann grade 2 without any sarcomatoid aspect and was treated with a nephrectomy. The tumour growth involved the renal pelvis without vascular invasion. Lymph nodes were not removed. During the postsurgical follow-up of both tumour diseases of more than five years, the follow-up examinations were unremarkable without any hints for tumour recurrence. The last follow-up of the RCC was one year before the patient noticed the preauricular tumour.

**Figure 1:**
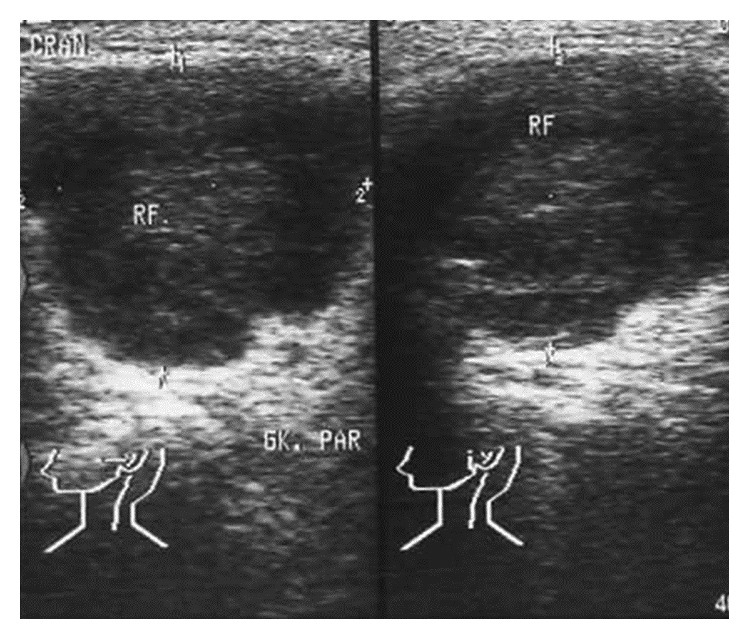
Ultrasonography of the left parotid gland.

The clinical examination showed a mobile lesion in the left parotid region. Ultrasound examination revealed a 3 cm polygonal hypoechoic lesion with well-defined smooth margins (Figure 1) so that our preoperative differential diagnosis included both benign and malignant neoplasms. Fine needle aspiration was not performed. The patient underwent parotidectomy with preservation of the facial nerve.

**Figure 2:**
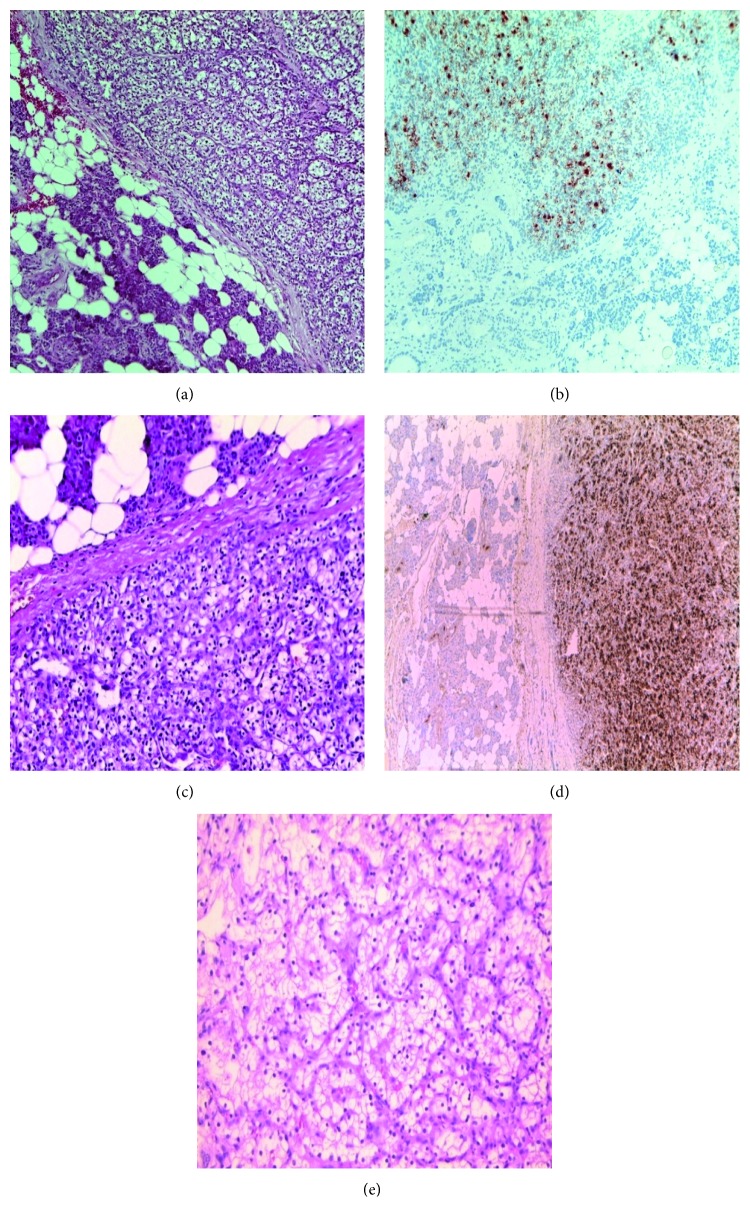
Microscopic image of the tumour of the left parotid gland (a, b), right parotid gland (c, d), and renal cancer (e).

Examination of the lesion showed a yellow tumour surrounded by a fibrous pseudocapsule, embedded in normal parenchyma. Histology showed monomorphous cells with serous-type clear cell cytoplasm, hyperchromatic nuclei, and partially acinus cell-like growth pattern of the parotid gland resembling acinic cell carcinoma. Differential diagnoses included primary clear cell carcinoma of the parotid gland, metastatic disease of the thyroid gland and the kidney including subtypes (clear cell carcinoma, papillary carcinoma, and chromophobe carcinoma), renal cell adenoma, lymphoma, sarcoma, oncocytoma, and mesenchymal tumours like schwannoma and angioleiomyoma. The histopathological diagnosis of RCC was based on the morphology and additional immunohistological analyzes. The tumour tissue was weakly diffuse granular PAS-positive, no evidence of PAS-positive mucous vacuoles. The epithelial membrane antigen showed positive, vigorous staining on approximately 80% of the tumour cells. The tumour cells showed a positive reaction to CK 10, CK 8, vimentin, and the renal cell carcinoma marker. Cytokeratin, carcinoembryonal antigen, p63, and S100 were negative. The proliferation index Ki 67 was below 10%. Therefore, the immunohistological staining presented a metastasis of a clear cell renal cell carcinoma and was consistent in its immunohistochemical pattern with the previously treated renal cell carcinoma (Figure 2).

Tumour restaging two years later revealed three small lesions in the lung and another three years later a mass in the liver that remained nearly constant in size during follow-up. Six years after parotidectomy of the left parotid gland, the patient presented with a mass of 1.5 cm in diameter of the right parotid gland. Parotidectomy was performed, and the mass was proved to be another metastasis of the renal cell carcinoma.

## 3. Discussion

Primary tumours metastasizing to the parotid gland are commonly located in the head and neck, with skin being the most frequent site (60%). Metastases from infraclavicular tumours (10–20%) are rare. Renal cell carcinoma (RCC) is slow growing and may be asymptomatic for a long time. At the time of diagnosis, 20% have locoregional or distant metastases, and in a further 30% of all RCC, metastases will occur following curative treatment. RCC would commonly spread to the lung and lymph nodes and also to bone and liver—in most cases, multiple organ systems are involved at once [3]. RCC are the third most common infraclavicular tumour with a pattern of metastasizing to the head and neck. In most cases, they will spread to the neck lymph nodes, but manifestations in paranasal sinus, skull, oral cavity, skin, larynx, and thyroid gland have been reported [2–4]. RCC metastasizing to the parotid gland has been presented by case reports or case series [2, 4–6]. To our knowledge, this is the first case report presenting the case of a patient with metastases of an RCC which affected both parotid glands six and twelve years following curative therapy.

In up to 50% of cases, supraclavicular metastasis may be the first manifestation of an RCC [4]. In other cases, they occur many years after therapy of the primary tumour and are thought to occur via both haematogenous and lymphogenous routes [2, 3, 5]. Metastases in the parotid gland usually present as painless, rapidly growing lesions without involvement of the facial nerve. They appear as hypoechoic, smooth-edged lesions on ultrasonography. A clue to their origin is enhanced vascularity seen on colour Doppler ultrasonography. If metastasis of an RCC is suspected, cross-sectional imaging is indicated to assess the primary tumour and exclude further metastases [2, 3, 5]. In cases where definitive histological classification is difficult, the algorithmic approach as proposed by Udager and Rungta using a limited set of immunohistological stains (pancytokeratin, vimentin, calponin, and p63) seems to be more sensible [7]. When parotid surgery is performed, a tendency to bleed is observed which reflects increased vascularisation [2, 5].

Current guidelines for metastatic RCC recommend surgical management wherever possible [7, 8]. Good oncological results have been achieved for solitary lung and liver metastases after successful therapy of the primary RCC [3]. As observed in our patient, the time elapsed between therapy of the primary tumour and appearance of the metastasis appeared to be an important prognostic factor [3]. However, supraclavicular metastasis can often be an expression of generalised tumour spread, and surgical treatment of RCC metastases is sometimes indicated for symptom control, for example, for pain control, haemorrhage, or impending airway obstruction [5].

## References

[1] Ellies G. L., Auclair P. L. (2008). *Secondary Tumors in Tumors of the Salivary Glands*.

[2] Park Y. W., Hlivko T. J. (2002). Parotid gland metastasis from renal cell carcinoma. *Laryngoscope*.

[3] Ljungberg B., Cowan N. C., Hanbury D. C. (2010). EAU guidelines on renal cell carcinoma: the 2010 update. *European Urology*.

[4] Langille G., Taylor S. M., Bullock M. J. (2008). Metastatic renal cell carcinoma to the head and neck: summary of 21 cases. *Journal of Otolaryngology-Head and Neck Surgery*.

[5] Deeb R., Zhang Z., Kini S., Ghanem T. (2010). Metastatic renal cell carcinoma to the parotid gland presenting 19 years after nephrectomy: case report and review of literature. *Laryngoscope*.

[6] Gottlieb M. D., Roland J. T. (1998). Paradoxical spread of renal cell carcinoma to the head and neck. *Laryngoscope*.

[7] Moch H., Cubilla A. L., Humphrey P. A., Reuter V. E., Ulbright T. M. (2016). The 2016 WHO classification of tumours of the urinary system and male genital organs-part a: renal, penile, and testicular tumours. *European Urology*.

[8] Udager A. M., Rungta S. A. (2014). Metastatic renal cell carcinoma, clear cell type, of the parotid gland: a case report, review of literature, and proposed algorithmic approach to salivary gland clear cell neoplasms in fine-needle aspiration biopsies. *Diagnostic Cytopathology*.

